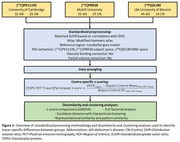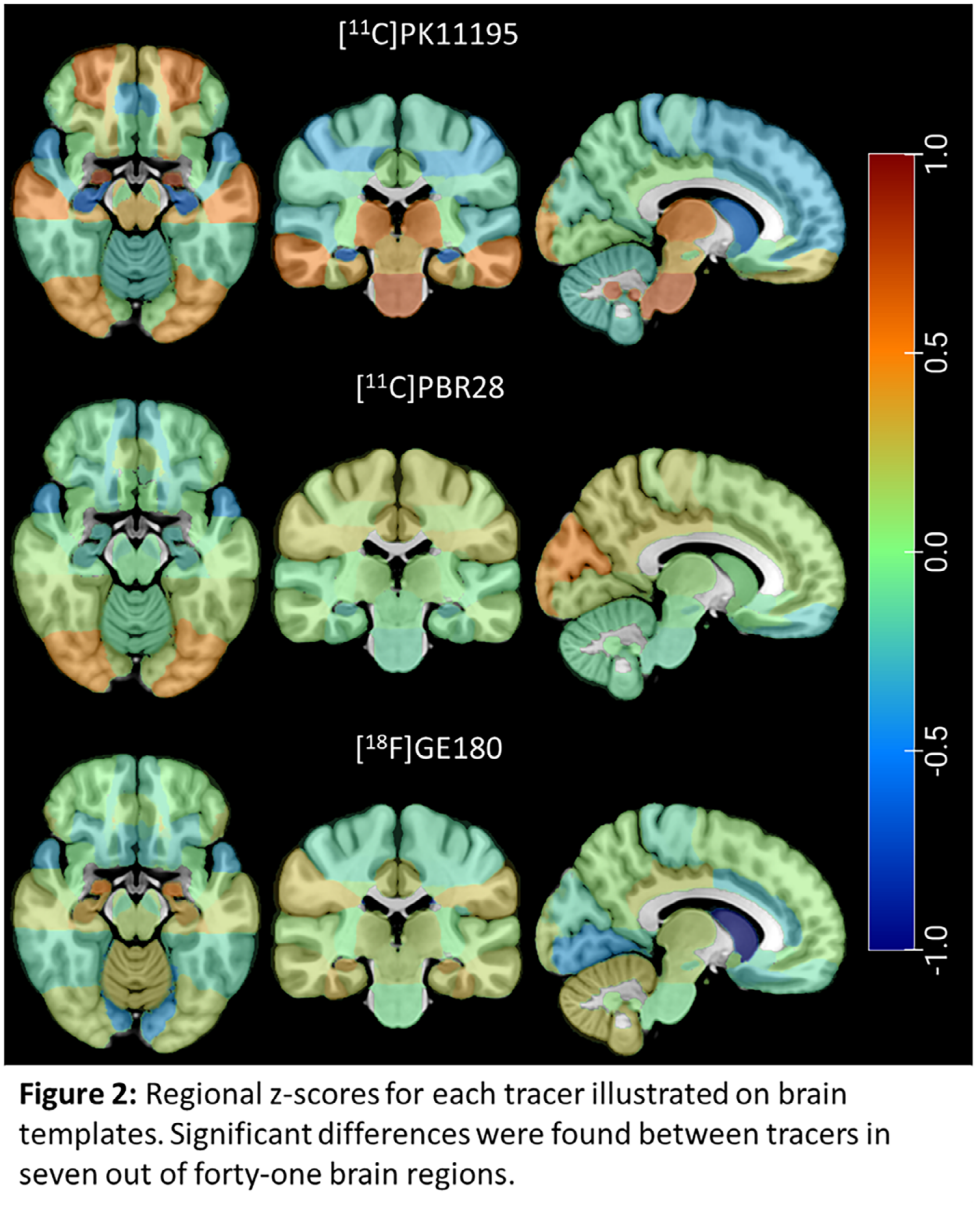# A pipeline for comparing and combining TSPO‐PET tracers in Alzheimer's disease

**DOI:** 10.1002/alz70856_104920

**Published:** 2026-01-07

**Authors:** Harry Crook, Nicolai Franzmeier, Nesrine Rahmouni, Johannes Gnörich, Alexandra Strauss, Sebastian Roemer‐Cassiano, Carla Palleis, P Simon Jones, Tim D Fryer, Young T Hong, Franklin I Aigbirhio, Johannes Levin, Günter U Höglinger, James B Rowe, John T O'Brien, Pedro Rosa‐Neto, Matthias Brendel, Maura Malpetti

**Affiliations:** ^1^ University of Cambridge, Cambridge, United Kingdom; ^2^ UK Dementia Research Institute at the University of Cambridge, Cambridge, United Kingdom; ^3^ University of Gothenburg, The Sahlgrenska Academy, Institute of Neuroscience and Physiology, Psychiatry and Neurochemistry, Gothenburg, Sweden; ^4^ Institute for Stroke and Dementia Research (ISD), University Hospital, LMU Munich, Munich, Bavaria, Germany; ^5^ Institute for Stroke and Dementia Research (ISD), University Hospital, LMU, Munich, Bayern, Germany; ^6^ Munich Cluster for Systems Neurology (SyNergy), Munich, Bavaria, Germany; ^7^ Montreal Neurological Institute, Montréal, QC, Canada; ^8^ Translational Neuroimaging Laboratory, The McGill University Research Centre for Studies in Aging, Montréal, QC, Canada; ^9^ Department of Nuclear Medicine, University Hospital, LMU Munich, Munich, Germany, Munich, Germany; ^10^ University Hospital, LMU Munich, Munich, Germany; ^11^ Department of Neurology, University Hospital, LMU Munich, Munich, Bavaria, Germany; ^12^ Max Planck School of Cognition, Leipzig, Sachsen, Germany; ^13^ German Center for Neurodegenerative Diseases (DZNE), Munich, Germany; ^14^ Munich Cluster for Systems Neurology (SyNergy), Munich, Germany; ^15^ Wolfson Brain Imaging Centre, University of Cambridge, Cambridge, United Kingdom; ^16^ University Hospital, LMU Munich, Munich, Bavaria, Germany; ^17^ Munich Cluster for Systems Neurology (SyNergy), Munich, Munich, Germany; ^18^ Ludwig‐Maximilians‐Universität München, Munich, Germany; ^19^ German Center for Neurodegenerative Diseases (DZNE), Munich, Bavaria, Germany; ^20^ Hannover Medical School, Hannover, Germany; ^21^ Cambridge University Hospitals NHS Foundation Trust, Cambridge, United Kingdom; ^22^ University of Cambridge, Cambridge, ‐, United Kingdom; ^23^ MRC Cognition and Brain Sciences Unit, University of Cambridge, Cambridge, United Kingdom; ^24^ Dementias Platform UK, Cambridge, United Kingdom; ^25^ Douglas Mental Health University Institute, Montreal, QC, Canada; ^26^ McConnell Brain Imaging Centre, Montreal Neurological Institute, McGill University, Montreal, QC, Canada; ^27^ McGill University, Montreal, QC, Canada; ^28^ Montreal Neurological Institute, Montreal, QC, Canada; ^29^ LMU University Hospital, Munich, Germany; ^30^ University of California, San Francisco, San Francisco, CA, USA

## Abstract

**Background:**

Neuroinflammation is a key pathological driver of neurodegenerative diseases, including Alzheimer's disease (AD). Positron emission tomography (PET) with tracers targeting the translocator protein (TSPO) enables the in vivo quantification of microglial activation. Currently, direct comparison between TSPO‐PET tracers in AD have not been performed. Here, we tested a pipeline to quantitatively compare different TSPO‐PET tracers in clinically‐matched cohorts of patients with AD across multi‐centre data.

**Method:**

32 people with AD and 15 controls underwent [^11^C]PK11195‐PET at the University of Cambridge, 45 people with AD and 19 controls underwent [^18^F]GE180‐PET at Ludwig‐Maximilians‐University of Munich, and 25 people with AD and 25 controls underwent [^11^C]PBR28‐PET at McGill University. Participants across the centres were matched for age, sex, and clinical severity. Pre‐processing of scans was harmonised across centres, and regional SUVr of tracers were obtained using a shared reference region and atlas. Z‐scores of regional SUVr values for each participant were calculated based on centre‐specific controls. Dissimilarity and clustering analyses were performed to assess the effectiveness of the standardisation pipeline. Figure 1 outlines the methodology.

**Result:**

Clustering analyses identified no tracer‐specific patterns in the distribution of z‐scores following standardisation. Across all tracers, regional z‐scores of the AD groups were significantly different between tracers in 7 of 41 brain regions, while no differences were found for controls (Figure 2). Full factorial analysis found a main effect of tracer; however, these were due to interaction effects with disease group, sex, age, and brain region and explained very little of the variance. Pattern similarity between representational similarity matrices found moderate correlations between the three tracers in patient and control groups.

**Conclusion:**

These results suggest that our pipeline is effective at harmonising TSPO‐PET tracers and standardising the regional quantification of microglial activation in the context of different AD cohorts. Dissimilarity analyses identified small tracer‐specific effects, however. Ongoing work aims to optimize this pipeline in order to compare and combine TSPO‐PET tracers in other tauopathies (ie PSP) and to identify thresholds of “inflammation severity” related to clinical outcomes.